# Supplementary material for: Flexibility and Hydration of the Qo Site Determine Multiple Pathways for Proton Transfer in Cytochrome bc 1
Source: J Chem Inf Model. 2025 Jun 10;65(12):6184–97. doi: 10.1021/acs.jcim.5c00655 (PMC12199298; doi:10.1021/acs.jcim.5c00655)
Supplement: Supplementary file 1 [file ci5c00655_si_001.pdf]

Supporting Information

Flexibility and hydration of the Q_o site determine multiple pathways for proton transfer in cytochrome bc_1

Sofia R. G. Camilo and Guilherme M. Arantes*

Department of Biochemistry, Instituto de Química, Universidade de São Paulo, Av. Prof. Lineu Prestes 748, 05508-900, São Paulo, SP, Brazil

E-mail: `garantes@iq.usp.br`

Contents:

- Supplementary Text: Details of bc_1 experimental structures analyzed here
- Supplementary Text: Altered force-field parameters for heme b and Rieske FeS ligands
- Table S1: Experimental structures analysed here
- Table S2: Force-field parameters for heme b and Rieske FeS ligands
- Figure S1: Root mean square deviations (RMSD) from MD simulations
- Figure S2: Root mean square fluctuations (RMSF) from MD simulations
- Figure S3: Convergence of metadynamics simulations and free energy profiles
- Figure S4: Trajectories of contacts by heme b_L A-propionate and by H276
- Figure S5: Trajectories of additional contacts bridged by water
- Figure S6: Comparison for interactions in the Q_o site with high-level quantum chemical methods
- Supporting References

Details of bc_1 experimental structures analyzed here

In order to probe the conformational distribution of residues in the Q_o site using high-resolution experimental data, 52 entries of cytochrome bc_1 and its b_6f analogue were retrieved from the PDB with a resolution better than 4.0Å. An initial set was obtained in 2021 when this analysis was first carried out. Various cryo-EM structures containing the bc_1 dimer were obtained more recently and all structures containing a bound Q substrate were added to this analysis resulting in 79 models of the Q_o site.

The Q_o experimental structures were classified into five conformational modes based on the side chains of Y147, E295, and Y297 (Fig. 10 and Table 1). Modes A and B together consist of 75 Q_o site models from various organisms. In Mode A, there are 51 structures predominantly from *Bos taurus* (12), *Saccharomyces cerevisiae* (10), *Arabidopsis thaliana* (6), *Mus musculus* (4), *Vigna radiata* (4), and *Sus scrofa* (4). In Mode B, 24 structures come primarily from *R. sphaeroides* (10), *Gallus gallus* (4), and *Saccharomyces cerevisiae* (4). The majority of these structures belong to the cytochrome bc_1 complex, with 50 structures alone, 22 as part of supercomplexes containing bc_1, and 3 of cytochrome b_6f. 55 models do not have resolved water molecules. Cryo-EM was used to determine 38 of these structures, while X-ray crystallography was used for 37. Regarding ligand presence at the Q_o site, 27 structures in Mode A have various ligands, while 21 structures in Mode B specifically have stigmatellin-A as a ligand, which is consistent with its role in inducing conformational changes at E295.[4]

In the mode C, there are only two structures from *Bos taurus* determined by X-ray crystallography without resolved water molecules. One structure contains the ligand *6-hydroxy-5-undecyl-1,3-benzothioazole-4,7-dione* (UHD). Mode D consists of only one structure of the cytochrome b_6f from *Chlamydomonas reinhardtii* determined by X-ray crystallography and does not contain resolved water molecules. The ligand at the Q_o site is *8-hydroxy-5,7-dimethoxy-3-methyl-2-tridecyl-4H-chromen-4-one* (TDS). Mode E also includes only one structure of the cytochrome b_6f complex obtained from *Mastigocladus laminosus*. This structure does not contain resolved water molecules or ligand at the Q_o site.

We identified 13 experimental models of the Q_o site occupied by Q (PDB entries: 1ntz, 6q9e, 7rja, 8asi, 8bel, 8bpx, 8bq5, 8bq6, 8e7s[13–19,83]), with one site occupied by ubiquinone-2 (UQ_2), four by UQ_5, two by UQ_6, four by UQ_7 and two by U_{10}. All of these models have the Rieske domain near the Q_o site, with H152 close to the Q-headgroup (Table S1).

The following PDB entries were analyzed here (Table S1): 1kb9, 1kyo, 1l0n, 1ntk, 1ntz, 1p84, 1q90, 1sqb, 1sqp, 1sqq, 1sqv, 1sqx, 2d2c, 2e76, 2fyn, 2fyu, 2ibz, 2qjk, 2qjp, 2qjy, 2ybb, 2yiu, 3h1i, 3h1j, 3l72, 4h13, 4pd4, 5kli, 5klv, 5nmi, 6fo6, 6hu9, 6kls, 6nhg, 6q9e, 6rqf, 6t0b, 7jrg, 7jrp, 7o37, 7o3c, 7o3e, 7o3h, 7rja, 7tlj, 8asi, 8bel, 8bpx, 8bq5, 8bq6, 8e7s, 8uge, 8ugf, 8ugg.[9,14–17,19,59,78,83–105]

Calibrated force-field parameters for heme b and Rieske FeS ligands

In cytochrome bc_1, heme b and heme c differ in their iron coordination. Heme b is coordinated by two His side chains, whereas heme c is coordinated by one His and one Met side chain. Additionally, the vinyl side chains in heme b are replaced by thioether linkages in heme c. The FeS cluster is bound to the Rieske protein by two Cys and two His side chains, an unsual coordination among FeS binding proteins.

Oxidized heme b groups were described here using the parameters obtained by Luthey-Schulten *et al.*[51] Both His side chains coordinated to iron were modeled with the bound His parameters.[51] The vinyl side chains in heme b employed standard CHARMM atom types. Partial charges for the vinyl carbon atoms were slightly adjusted: $-0.02|e|$ for CA, and a redistribution of $0.09|e|$ to CB from the removed HB (Table S2). Propionate side chains in both hemes employed standard CHARMM atom types and parameters. These CHARMM parameters should

provide a balanced treatment of interactions for heme *b* groups.

For both Cys and His ligands bound to the Rieske [2Fe-2S] cluster, the atom types (with associated Lennard-Jones and bonded force-field parameters) proposed by Chang and Kim[50] were employed. These FeS parameters have been used extensively in previous MD simulations of cytochrome bc_1[26,69] and other respiratory complexes containing [2Fe-2S] clusters.[64,106,107] Partial charges for the side chains were re-calibrated (Table S2), particularly for His152 which was modeled in its deprotonated form in our MD simulations. The monoanionic compound [2Fe-2S](S-$CH_2CH_3)_2(ImH)(Im)^-$, where ImH is 4-methylimidazole and Im is the (N_ϵ-)deprotonated 4-methylimidazolium, was used as a model. Quantum-chemical (QC) calculations with ferromagnetic (high-spin) coupling at the B3LYP/6-31G(d) level of theory[108–110] with Merz-Kollmann partial charges[111] were taken as a reference.

The accuracy of the molecular mechanics (MM) parametrization for both heme *b* and FeS center with its H152 ligand was evaluated by comparing potential energies calculated using quantum chemical methods for relevant interactions in the Q_o site (Fig. S6A). Relative energies were computed using a high-level quantum chemical method, MP2,[112] with the def2-TZVP basis set[113] – a benchmark for force-field calibration[48] – and compared to the standard hybrid DFT functional B3LYP with dispersion corrections (D3)[114] and the 6-31G(d) basis set, as well as the classical MM force-field used for MD simulations. Energies were calculated for the same set of relaxed geometries, optimized at the MM level by scanning the atom-pair distances labeled on the X-axis in Fig. S6B-D. A hybrid QM/MM partitioning scheme was employed for the quantum chemical calculations.[69] The `pDynamo` library (version 3),[115] interfaced with `ORCA` (version 5),[116] was used for these calculations. The initial structure of the Q_o site was retrieved from our MD simulations (at 141 ns of the metadynamics simulation activated in chain D of the cyt *b* subunit) and is available online,[117] along with sample scripts to allow full reproduction of these comparisons.

Fig. S6B-D shows that the force-field accurately describes the interactions between PRA_{bL} and water, H152 and water, and H152 and QH_2 in the Q_o site, with a mean unsigned error of only 4.5 kJ/mol compared to the MP2 benchmark. The B3LYP method, an accurate and widely used standard in computational chemistry, performs only slightly better, with a mean unsigned error of 3.8 kJ/mol. Notably, B3LYP performs worse than the force-field for the interaction between PRA_{bL} and water (Fig. S6B). Thus, the molecular mechanics force-field parametrization used here is accurate and effectively describes key interactions in the Q_o site, supporting our MD simulation results and conclusions.

Table S1: Experimental structures analysed here (N=79). Column PDB gives the entry code. Ligand is the small-molecule ligand found in the Q_o site, denoted with the PDB identification. Distance (in Å) is the separation between cyt *b* Y147 C_β and Rieske H152 N_ϵ, and Mode is the torsional mode of YEY side-chains as identified in Table 1.

PDB	Ligand	Distance	Mode	PDB	Ligand	Distance	Mode
2e76	TDS	14.8	A	8bpx	UQ7	14.9	A
1l0n		15.5	A	8bq5	UQ5	15.2	A
1ntz	UQ2	15.6	A	8bq5	UQ7	15.1	A
1p84	DBT	15.0	A	8bq6	UQ5	15.0	A
1sqb	AZO	17.8	A	8bq6	UQ7	15.1	A
1sqp	MYX	17.5	A	8e7s	UQ6	15.3	A
1sqq	OST	17.9	A	8e7s	UQ6	17.1	A
2fyu	FDN	16.0	A	8uge		18.2	A
3l72	IKR	23.8	A	8uge		28.7	A
3l72	IKR	24.0	A	8ugg		27.1	A
4h13	TDS	14.5	A	8ugg		28.2	A
4pd4	AOQ	14.9	A	1kb9	SMA	15.1	B
5klv	FNM	16.0	A	1kyo	SMA	15.1	B
5nmi		-	A	1kyo	SMA	15.2	B
5nmi		20.0	A	1sqx	SMA	14.9	B
6fo6		-	A	2fyn	SMA	14.8	B
6fo6		-	A	2fyn	SMA	15.1	B
6hu9		33.0	A	2ibz	SMA	15.1	B
6hu9		24.9	A	2qjk	SMA	14.9	B
6kls		27.7	A	2qjk	SMA	15.0	B
6kls		27.8	A	2qjp	SMA	15.0	B
6nhg	AZO	17.9	A	2qjp	SMA	15.0	B
6rqf		17.7	A	2qjy	SMA	14.9	B
6t0b		24.6	A	2qjy	SMA	14.7	B
6t0b		33.3	A	2ybb	SMA	14.9	B
7jrg		-	A	2ybb	SMA	14.8	B
7jrg		-	A	2yiu	SMA	14.8	B
7jrp		-	A	2yiu	SMA	14.8	B
7jrp		-	A	3h1i	SMA	15.2	B
7o37		30.8	A	3h1i	SMA	15.1	B
7o3c		28.1	A	3h1j	SMA	15.1	B
7o3c		27.2	A	3h1j	SMA	15.3	B
7o3h		27.3	A	5kli	SMA	14.8	B
7rja	U10	18[a]	A	5kli	SMA	14.9	B
7tlj	PQU	16.1	A	8ugf		28.7	B
7tlj	PQU	16.3	A	1ntk		16.1	C
8asi	U10	15.1	A	1sqv	UHD	15.8	C
8bel	UQ5	15.1	A	1q90	TDS	15.4	D
8bel	UQ7	14.9	A	2d2c		18.4	E
8bpx	UQ5	15.1	A				

The Y147–H152 distance is less than 20Å in 56 of the 79 strucutures, indicating that the Rieske head domain, with H152 and the FeS center, is in close contact to the Q_o site. A "-" in the Distance column indicates missing residues or incomplete structure in the PDB model. [a] Estimated due to lack of H152 side-chain.

Table S2: Force-field parameters altered from references[50,51] and used here for heme *b* and side chains bound to the Rieske FeS cluster.

Atom name	Atom type	Partial charge
His131 bound to FeS:		
CB	CT2	0.004
HB	HA2	0.090
ND1	NR2	-0.111
CG	CPH1	-0.096
CE1	CPH2	0.012
HE1	HR1	0.156
NE2	NR1	-0.184
HE2	H	0.337
CD2	CPH1	-0.159
HD2	HR3	0.195
His152 bound to FeS:		
CB	CT2	-0.100
HB	HA2	0.090
ND1	NR2	-0.400
CG	CPH1	-0.100
CE1	CPH2	0.000
HE1	HR1	0.100
NE2	NR1	-0.350
CD2	CPH1	-0.096
HD2	HR3	0.100
Cys129 and Cys149 bound to FeS:		
CB	CT2	-0.65973
Heme *b*:		
CA	CE1	-0.360
HA	HE1	0.160
CB	CE2	-0.160
HB	HE2	0.090

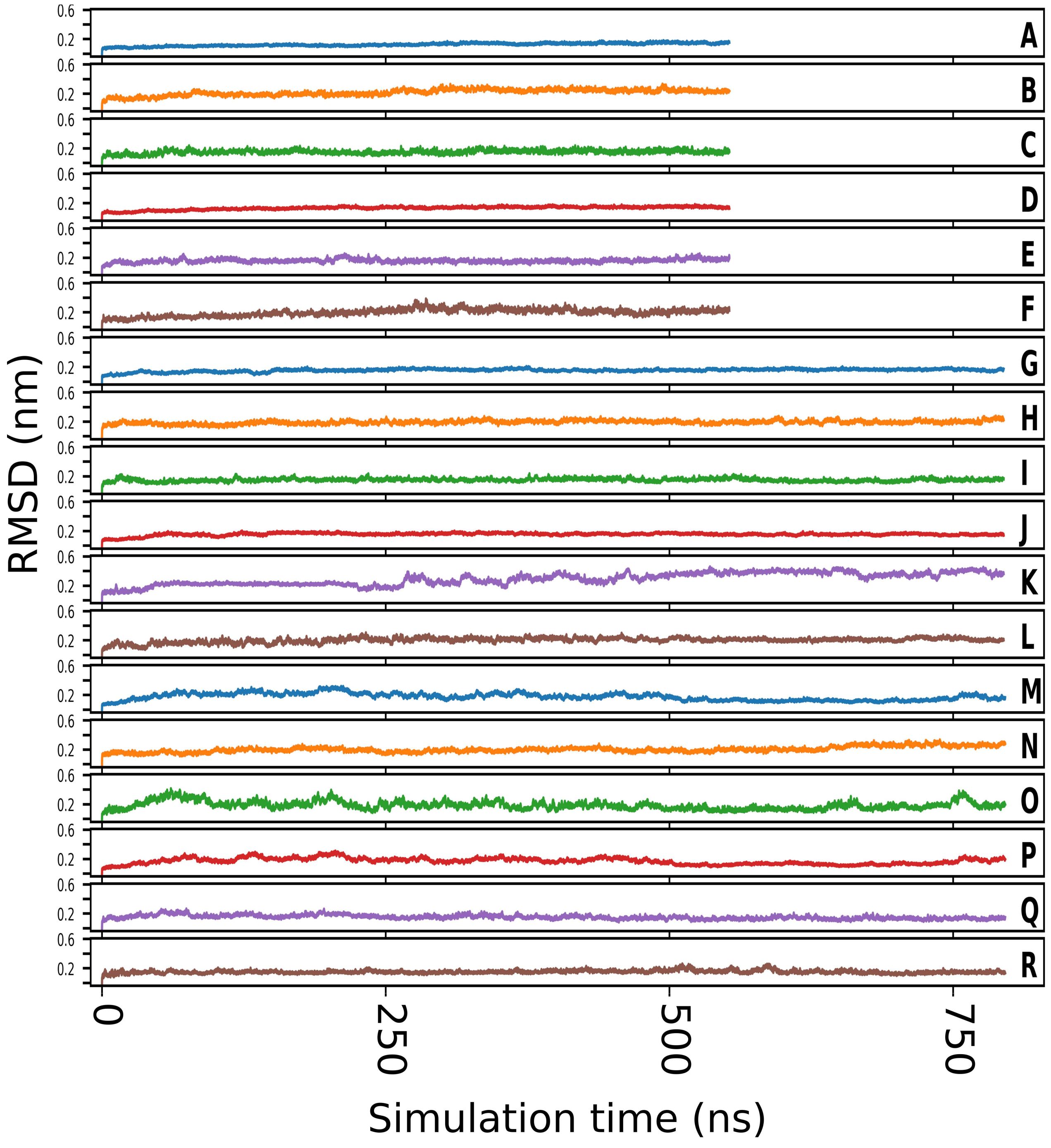

Figure S1: Root mean square deviations (RMSD) of Cα atoms obtained from MD simulations of the cytochrome bc_1 dimer, for each protein subunit named on top of each column. **A-F** show the 550 ns canonical MD. **G-L** have metadynamics activated for the YEY group of the Q_o site in chain (1)-Cyt b. **M-R** has metadynamics activated for the YEY group in chain (2)-Cyt b. Colors code for protein subunit as in Fig. S2. All simulations remain stable and only show normal fluctuations for this time-scale, connected to flexibility in terminal or unstructured regions. For instance, the transition observed in panel **K** for Cyt c_1 around 300-500 ns corresponds to residues 185 to 220 (high RMSF in Fig. S2B), a flexible region exposed to solvent and without stable secondary structure.

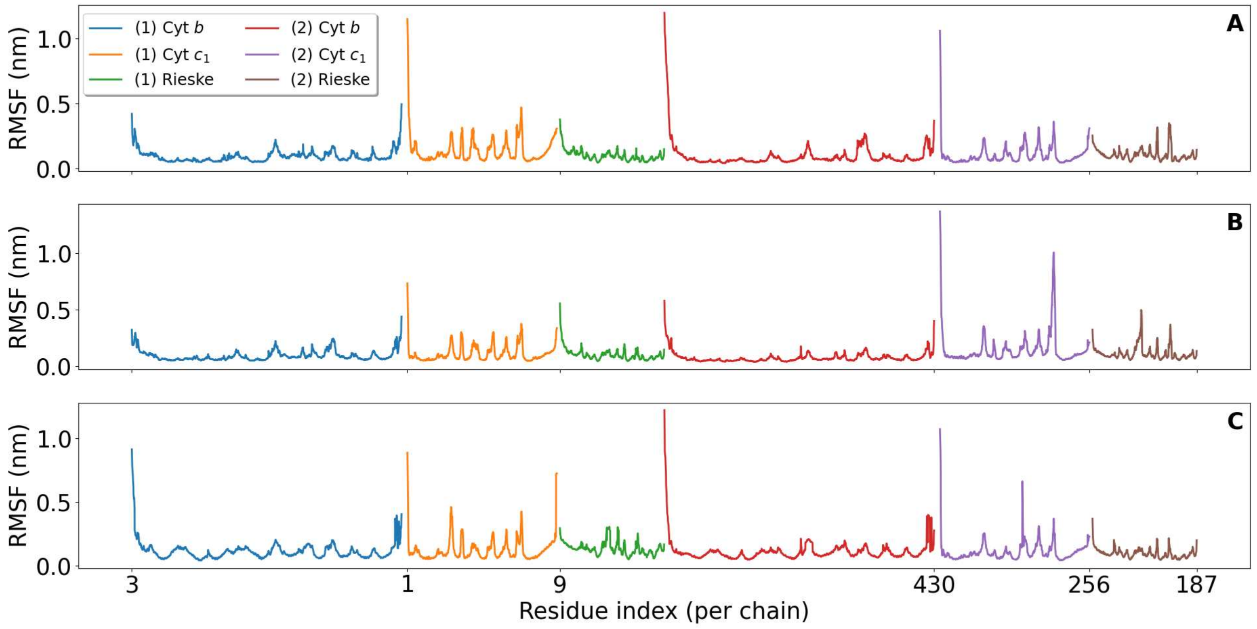

Figure S2: Root mean square fluctuations (RMSF) of backbone heavy-atoms obtained from MD simulations of the cytochrome bc_1 dimer. **A** shows the 550 ns canonical MD. **B** has metadynamics activated for Q_o site in chain (1)-Cyt b. **C** has metadynamics activated for Q_o site in chain (2)-Cyt b. Initial and final residue indexes are shown for subunit sets (1) and (2), respectively.

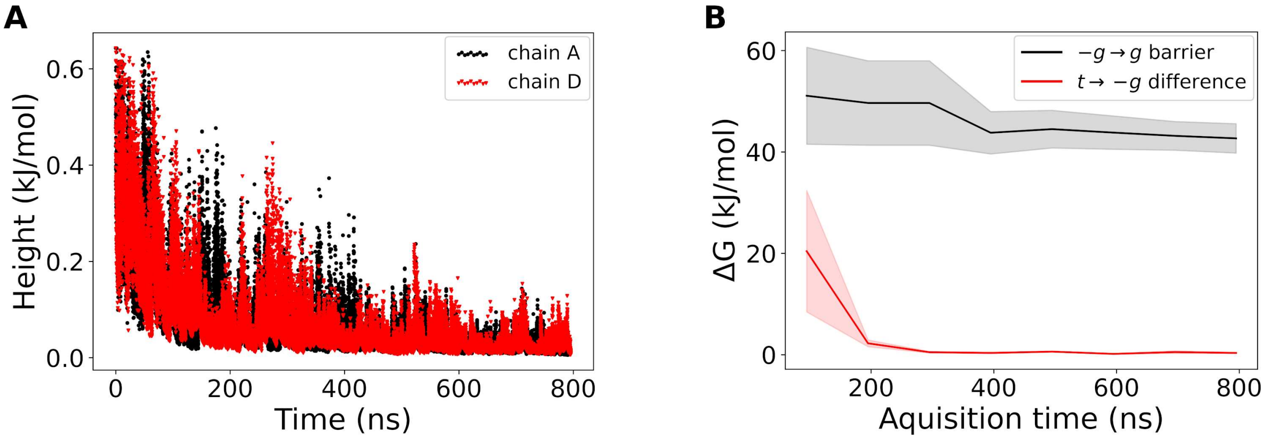

Figure S3: Convergence of the metadynamics simulations and derived free energy profiles. **A** shows the time evolution of the gaussian height in the two well-tempered metadynamics simulations performed (Fig. 3). The height decreases and levels off around 400 ns, suggesting that a smoother energy surface is visited and enhanced sampling of the YEY side chain dihedrals is achieved. **B** shows the convergence with simulation time of three free energy differences between $Y147_{\chi 1}$ conformations indicated in the legend. The average between the two simulations is shown with colored shadows giving the standard error. Variations in free energies are smaller than this statistical error after 400ns, indicating convergence. A similar behavior is observed in free energy profiles for the other boosted YEY dihedrals.

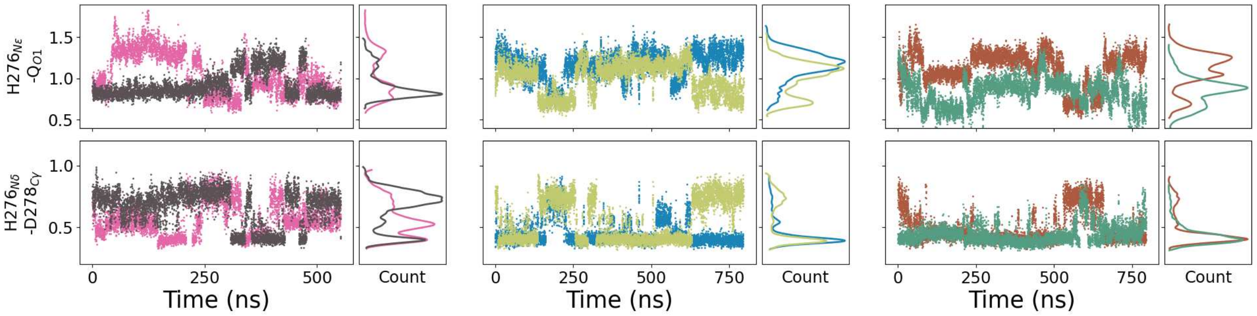

Figure S4: Atom-pair distances involving heme PRA_{bL} in **A**-**C** and H276 in **D** and **F**. Pairs are given in Y-axis labels. Panel columns and colors relate to the six MD simulations of the Q_o site as described in Fig. 4. Count shows a histogram of the respective distance.

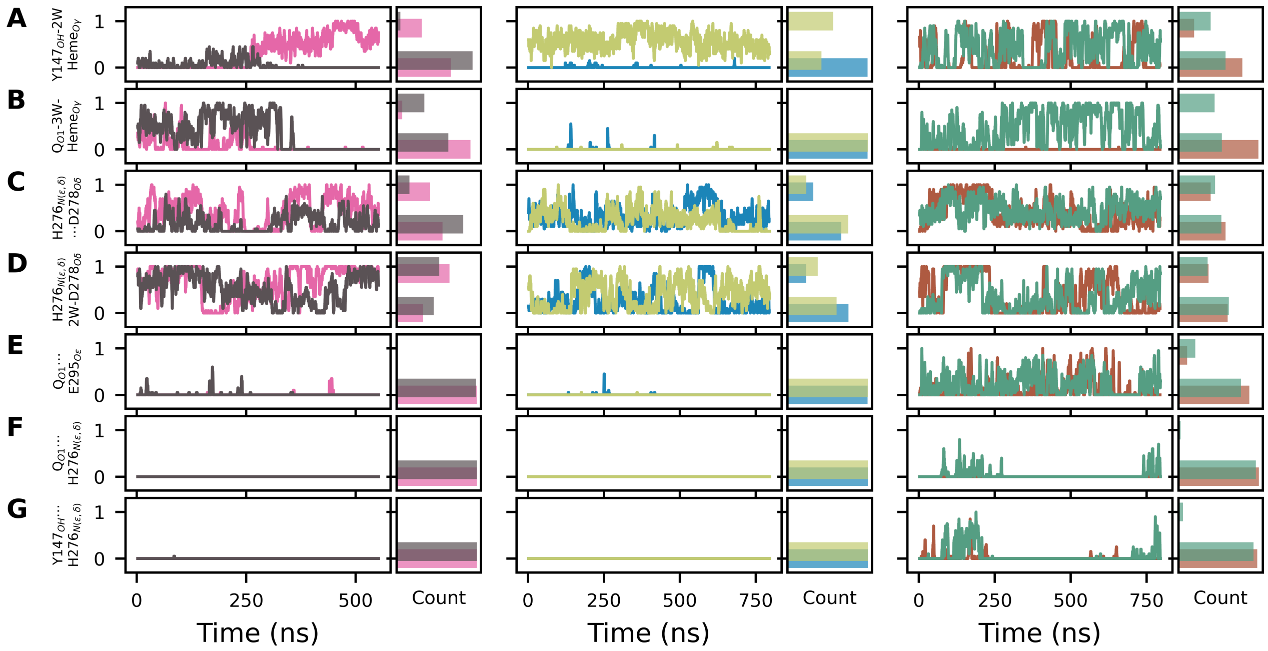

Figure S5: Additional contacts bridged by water, as labeled in Y-axis. Dots $(\cdots)$ in labels correspond to one bridge water molecule. Bridges with two or three waters are labeled 2W or 3W, respectively. One or zero correspond to the contact formed or not, respectively. A moving-average with a 1 ns window is plotted. Count shows a histogram of contact formation. Panel columns and colors relate to the six MD simulations of the Q_o site as described in Fig. 4.

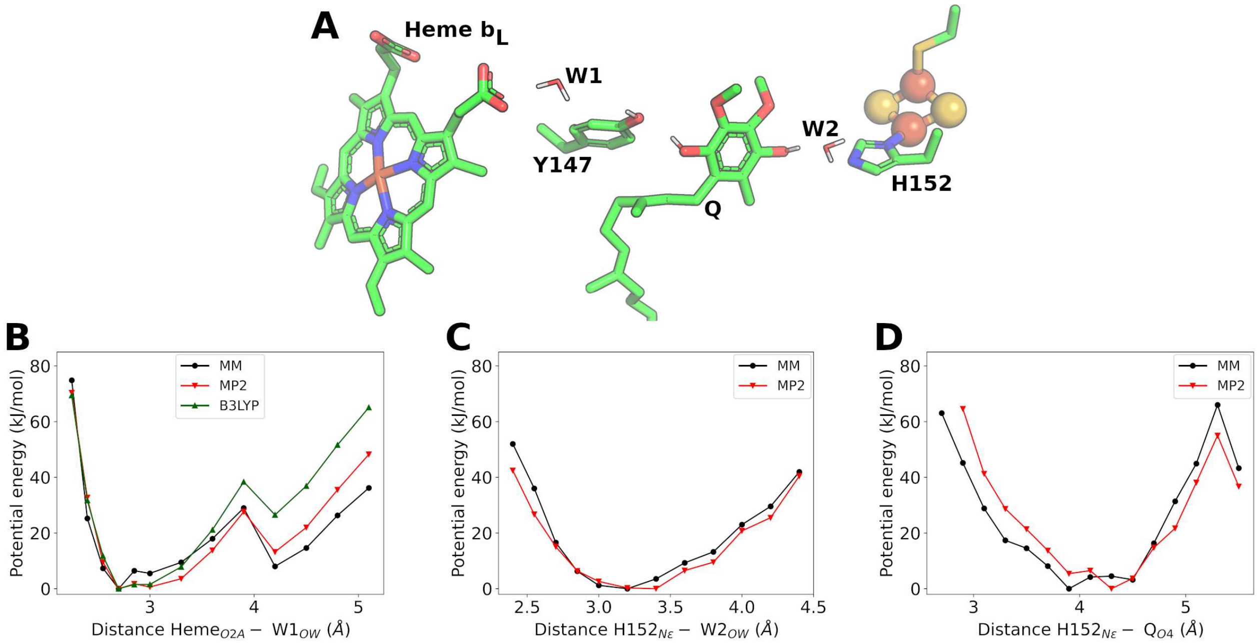

Figure S6: Comparison for relevant interactions in the Q_o site of potential energies calculated with high-level quantum chemical methods and the CHARMM-based parametrization of the molecular mechanics (MM) force-field used here. **A** shows the initial structure of the Q_o site used for comparisons. Only a few groups and polar hydrogens are shown for clarity, but the complete all-atom model of the Q_o site was used in calculations. Relative potential energies for interactions between are shown in **B** for PRA_{bL} and water (W1), **C** for H152 and water (W2), and **D** for H152 and QH_2. Atom pairs are indicated in the label of the X-axis. Energies were obtained at the MP2/def2-TZVP (MP2) and B3LYP-D3/6-31G(d) (B3LYP) levels of theory, as well as with the pure molecular mechanics (MM) force-field. For clarity, B3LYP profiles were not shown in panels **C** and **D** because they overlap with the other profiles.

Supporting References

(83) Gao, X.; Wen, X.; Esser, L.; Quinn, B.; Yu, L.; Yu, C.-A.; Xia, D. Structural Basis for the Quinone Reduction in the bc_1 Complex: A Comparative Analysis of Crystal Structures of Mitochondrial Cytochrome bc_1 with Bound Substrate and Inhibitors at the Qi Site. *Biochemistry* **2003**, *42*, 9067–9080.

(84) Lange, C.; Nett, J. H.; Trumpower, B. L.; Hunte, C. Specific roles of protein-phospholipid interactions in the yeast cytochrome bc_1 complex structure. *EMBO J.* **2001**, *20*, 6591–6600.

(85) Lange, C.; Hunte, C. Crystal structure of the yeast cytochrome bc_1 complex with its bound substrate cytochrome *c*. *Proc. Natl. Acad. Sci. USA* **2002**, *99*, 2800–2805.

(86) Palsdottir, H.; Lojero, C. G.; Trumpower, B. L.; Hunte, C. Structure of the Yeast Cytochrome bc_1 Complex with a Hydroxyquinone Anion Qo Site Inhibitor Bound. *J. Biol. Chem.* **2003**, *278*, 31303–31311.

(87) Stroebel, D.; Choquet, Y.; Popot, J.-L.; Picot, D. An atypical haem in the cytochrome b_6f complex. *Nature* **2003**, *426*, 413–418.

(88) Yan, J.; Kurisu, G.; Cramer, W. A. Intraprotein transfer of the quinone analogue inhibitor 2,5-dibromo-3-methyl-6-isopropyl-*p*-benzoquinone in the cytochrome b_6f complex. *Proc. Natl. Acad. Sci. USA* **2006**, *103*, 69–74.

(89) Yamashita, E.; Zhang, H.; Cramer, W. Structure of the Cytochrome b_6f Complex: Quinone Analogue Inhibitors as Ligands of Heme *c*. *J. Mol. Biol.* **2007**, *370*, 39–52.

(90) Esser, L.; Gong, X.; Yang, S.; Yu, L.; Yu, C.-A.; Xia, D. Surface-modulated motion switch: Capture and release of iron-sulfur protein in the cytochrome bc_1 complex. *Proc. Natl. Acad. Sci. USA* **2006**, *103*, 13045–13050.

(91) Lancaster, C. R. D.; Hunte, C.; Kelley, J.; Trumpower, B. L.; Ditchfield, R. A Comparison of Stigmatellin Conformations, Free and Bound to the Photosynthetic Reaction Center and the Cytochrome bc_1 Complex. *J. Mol. Biol.* **2007**, *368*, 197–208.

(92) Kleinschroth, T.; Castellani, M.; Trinh, C. H.; Morgner, N.; Brutschy, B.; Ludwig, B.; Hunte, C. X-ray structure of the dimeric cytochrome bc_1 complex from the soil bacterium Paracoccus denitrificans at 2.7-Å resolution. *Biochim. Biophys. Acta* **2011**, *1807*, 1606–1615.

(93) Zhang, Z.; Huang, L.; Shulmeister, V. M.; Chi, Y.-I.; Kim, K. K.; Hung, L.-W.; Crofts, A. R.; Berry, E. A.; Kim, S.-H. Electron transfer by domain movement in cytochrome bc_1. *Nature* **1998**, *392*, 677–684.

(94) Hasan, S. S.; Yamashita, E.; Baniulis, D.; Cramer, W. A. Quinone-dependent proton transfer pathways in the photosynthetic cytochrome b_6f complex. *Proc. Natl. Acad. Sci. USA* **2013**, *110*, 4297–4302.

(95) Birth, D.; Kao, W.-C.; Hunte, C. Structural analysis of atovaquone-inhibited cytochrome bc_1 complex reveals the molecular basis of antimalarial drug action. *Nat. Commun.* **2014**, *5*, 4029.

(96) Esser, L.; Zhou, F.; Zhou, Y.; Xiao, Y.; Tang, W.; Yu, C.; Qin, Z.; Xia, D. Hydrogen Bonding to the Substrate Is Not Required for Rieske Iron-Sulfur Protein Docking to the Quinol Oxidation Site of Complex III. *J. Biol. Chem.* **2016**, *291*, 25019–25031.

(97) McPhillie, M. et al. New paradigms for understanding and step changes in treating active and chronic, persistent apicomplexan infections. *Sci. Rep.* **2016**, *6*, 29179.

(98) Amporndanai, K.; Johnson, R. M.; O'Neill, P. M.; Fishwick, C. W. G.; Jamson, A. H.; Rawson, S.; Muench, S. P.; Hasnain, S. S.; Antonyuk, S. V. X-ray and cryo-EM structures of inhibitor-bound cytochrome bc_1 complexes for structure-based drug discovery. *IUCrJ* **2018**, *5*, 200–210.

(99) Hartley, A. M.; Lukoyanova, N.; Zhang, Y.; Cabrera-Orefice, A.; Arnold, S.; Meunier, B.; Pinotsis, N.; Maréchal, A. Structure of yeast cytochrome *c* oxidase in a supercomplex with cytochrome bc_1. *Nat. Struct. Mol. Biol.* **2019**, *26*, 78–83.

(100) Zhu, G.; Zeng, H.; Zhang, S.; Juli, J.; Pang, X.; Hoffmann, J.; Zhang, Y.; Morgner, N.; Zhu, Y.; Peng, G.; Michel, H.; Sun, F. 3.3Å -Resolution Structure of Hyperthermophilic Respiratory Complex III Reveals the Mechanism of Its Thermal Stability. *Angew. Chem. Int. Ed.* **2020**, *59*, 343–351.

(101) Esser, L.; Zhou, F.; Yu, C.-A.; Xia, D. Crystal structure of bacterial cytochrome bc_1 in complex with azoxystrobin reveals a conformational switch of the Rieske iron–sulfur protein subunit. *J. Biol. Chem.* **2019**, *294*, 12007–12019.

(102) Malone, L. A.; Qian, P.; Mayneord, G. E.; Hitchcock, A.; Farmer, D. A.; Thompson, R. F.; Swainsbury, D. J. K.; Ranson, N. A.; Hunter, C. N.; Johnson, M. P. Cryo-EM structure of the spinach cytochrome b_6f complex at 3.6Å resolution. *Nature* **2019**, *575*, 535–539.

(103) Hartley, A. M.; Meunier, B.; Pinotsis, N.; Maréchal, A. Rcf2 revealed in cryo-EM structures of hypoxic isoforms of mature mitochondrial III-IV supercomplexes. *Proc. Natl. Acad. Sci. USA* **2020**, *117*, 9329–9337.

(104) Maldonado, M.; Guo, F.; Letts, J. A. Atomic structures of respiratory complex III_2, complex IV, and supercomplex III_2-IV from vascular plants. *eLife* **2021**, *10*, e62047.

(105) Vercellino, I.; Sazanov, L. A. Structure and assembly of the mammalian mitochondrial supercomplex CIII2CIV. *Nature* **2021**, *598*, 364–367.

(106) Pereira, C. S.; Teixeira, M. H.; Russell, D. A.; Hirst, J.; Arantes, G. M. Mechanism of rotenone binding to respiratory complex I depends on ligand flexibility. *Sci. Rep.* **2023**, *13*, 6738.

(107) Gupta, C.; Khaniya, U.; Chan, C. K.; Dehez, F.; Shekhar, M.; Gunner, M. R.; Sazanov, L.; Chipot, C.; Singharoy, A. Charge Transfer and Chemo-Mechanical Coupling in Respiratory Complex I. *J. Am. Chem. Soc.* **2020**, *142*, 9220–9230.

(108) Becke, A. D. Density Functional Thermochemistry. III. The Role of Exact Exchange. *J. Chem. Phys.* **1993**, *98*, 5648.

(109) Lee, C.; Yang, W.; Parr, R. Development of the Colle-Salvetti Correlation-Energy Formula into a Functional of the Electron Density. *Phys. Rev. B* **1988**, *37*, 785–789.

(110) Ditchfield, R.; Hehre, W.; Pople, J. A. Self-Consistent Molecular-Orbital Methods. IX. An Extended Gaussian-Type Basis for Molecular-Orbital Studies of Organic Molecules. *J. Chem. Phys.* **1971**, *54*, 724–728.

(111) Field, M. J. *A Pratical Introduction to the Simulation of Molecular Systems*, 1st ed.; Cambridge University Press: Cambridge, 1999.

(112) Helgaker, T.; Jørgensen, P.; Olsen, J. *Molecular Electronic-Structure Theory*, 1st ed.; Wiley: New York, 2000.

(113) Weigend, F.; Ahlrichs, R. Balanced Basis Sets of Split Valence, Triple Zeta Valence and Quadruple Zeta Valence Quality for H to Rn: Design and Assessment of Accuracy. *Phys. Chem. Chem. Phys.* **2005**, *7*, 3297–3305.

(114) Grimme, S.; Antony, J.; Ehrlich, S.; Krieg, H. A Consistent and Accurate ab initio Parametrization of Density Functional Dispersion Correction (DFT-D) for the 94 Elements H-Pu. *J. Chem. Phys.* **2010**, *132*, 154104.

(115) Field, M. J. pDynamo3 Molecular Modeling and Simulation Program. *J. Chem. Inf. Model.* **2022**, *62*, 5849–5854.

(116) Neese, F. Software Update: The ORCA Program System, Version 4.0. *WIREs Rev. Comput. Mol. Sci.* **2018**, *8*, e1327.

(117) Arantes, G. M. Dataset: Redox-Activated Proton Transfer through a Redundant Network in the Q_o Site of Cytochrome bc_1. 2024; `https://doi.org/10.5281/zenodo.14198667`, Accessed: 2024-11-20.
